# Mitigating effects of *ex situ* application of rice straw on CH_4_ and N_2_O emissions from paddy-upland coexisting system

**DOI:** 10.1038/srep37402

**Published:** 2016-11-21

**Authors:** Wei Wang, Xiaohong Wu, Anlei Chen, Xiaoli Xie, Yunqiu Wang, Chunmei Yin

**Affiliations:** 1 Key Laboratory of Agro-ecological Processes in Subtropical Region, Institute of Subtropical Agriculture, Chinese Academy of Sciences, Changsha 410125, Hunan, China; 2 Faculty of Life Science and Technology, Central South University of Forestry and Technology, Changsha 410004, Hunan, China

## Abstract

The *in situ* application of rice straw enhances CH_4_ emissions by a large margin. The *ex situ* application of rice straw in uplands, however, may mitigate total global warming potential (GWP) of CH_4_ and N_2_O emissions from paddy-upland coexisting systems. To evaluate the efficiency of this practice, two field trials were conducted in rice-rice-fallow and maize-rape cropping systems, respectively. Year-round measurements of CH_4_ and N_2_O emissions were conducted to evaluate the system-scaled GWP. The results showed that CH_4_ accounted for more than 98% of GWP in paddy. Straw removal from paddy decreased 44.7% (302.1 kg ha^−1^ yr^−1^) of CH_4_ emissions and 51.2% (0.31 kg ha^−1^ yr^−1^) of N_2_O emissions, thus decreased 44.8% (7693 kg CO_2_-eqv ha^−1^ yr^−1^) of annual GWP. N_2_O accounted for almost 100% of GWP in upland. Straw application in upland had insignificant effects on CH_4_ and N_2_O emissions, which increased GWP only by 91 kg CO_2_-eqv ha^−1^ yr^−1^. So, the transfer of straw from paddy to upland could decrease GWP by 7602 kg CO_2_-eqv ha^−1^ yr^−1^. Moreover, straw retention during late rice season contributed to 88.2% of annual GWP increment. It is recommended to transfer early rice straw to upland considering GWP mitigation, nutrient recycling and labor cost.

Atmospheric methane (CH_4_) and nitrous oxide (N_2_O) are important greenhouse gases (GHG) causing global warming effect. The emissions of CH_4_ and N_2_O from agricultural soils accounted for approximately 50% and 60% of their corresponding global anthropogenic sources, respectively^1^. The gross global warming potential (GWP) of CH_4_ and N_2_O emissions was estimated to increase by up to 69% in crop land in China, from 244 Tg CO_2_-eqv (equivalent) yr^−1^ in the early 1980 s to 413 Tg CO_2_-eqv yr^−1^ in the late 2000 s^2^.

CH_4_ can be produced from biological decomposition of organic materials under anaerobic soil environment by methanogens, such as rice field under flooded conditions^3^,^4^. Paddy soils, accounting for the largest anthropogenic wetlands on earth, are among the most important sources of CH_4_. Currently, straw retention in paddy fields has become prevalent, mainly due to nutrient recycling and forbidden straw burning^5^,^6^,^7^,^8^. However, it provides abundant substrates and exerts priming effect on soil organic matter to release additional substrates for CH_4_ production, thus accelerating CH_4_ emissions by a large margin^7^,^9^,^10^,^11^. Also, with the increase of biomass, the amount of rice straw substantially exceeds the capacity for *in situ* incorporation, resulting in a large proportion (30~50%) burnt illegally by farmers^8^.

N_2_O can be generated primarily by the microbial processes of nitrification and denitrification in aerobic soil condition^12^,^13^. In contrast to paddy fields that primarily release CH_4_, upland soils primarily release N_2_O. It is estimated that upland croplands contributed most to national annual synthetic fertilizer N-induced direct N_2_O emissions, accounting for 79% in 1980 and 92% in 2000^14^. The *ex situ* application of rice straw in upland soils can improve soil organic carbon, nitrogen and microbial biomass^5^,^8^, which may enhance N_2_O emissions.

Uplands adjoin paddy fields in hilly areas in subtropical region of China, which forms typical paddy-upland coexisting agro-ecology system. Upland soils are usually characterized with low organic matter and nutrients, poor structure, high acidity, and low water retention^15^,^16^. A few studies have reported that transferring surplus rice straw from paddy system to upland system is an effective practice to improve organic matter, fertility, and productivity of upland soils^5^,^8^. Some studies investigated the *in situ* rice straw management on GHG emissions, and found that straw mulching, straw off-season application and straw-derived biochar application could decrease the GWP compared with the incorporation of fresh straw into soil^17^,^18^,^19^,^20^,^21^. However, the effectiveness of rice straw *ex situ* application on mitigation of CH_4_ and N_2_O has not been studied yet. Since the GWP of non-flooded crops is lower compared with that of flooded rice^22^, we hypothesized that the introduction of rice straw into uplands could not only facilitate nutrient recycling, but also greatly mitigate total GWP of CH_4_ and N_2_O emissions from the paddy-upland coexisting system. Thus, the aim of this study was to evaluate the effectiveness of *ex situ* application of rice straw in uplands to mitigate GHG emissions compared with the *in situ* application of rice straw in paddy fields. The investigation was carried out in two adjacent field trials in flooded rice-rice-fallow field and in maize-rape field in a hilly area in subtropical China. This would provide valuable knowledge for GHG mitigation and straw management in hilly paddy-upland coexisting system.

## Results

### CH_4_ and N_2_O emissions with *in situ* application of rice straw

**Figure 1:**
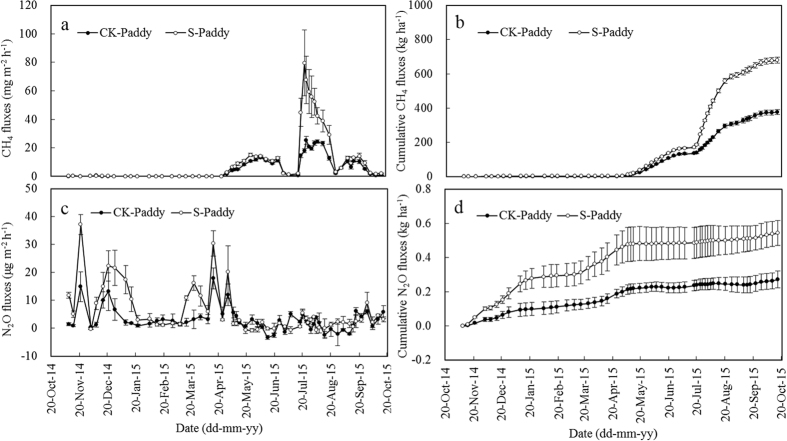
Dynamics of CH_4_ and N_2_O emissions from paddy as affected by *in situ* application of rice straw. S-Paddy means rice-rice-fallow system with straw retention at a rate of 5000 kg ha^−1^ season^−1^ and CK-Paddy means rice-rice-fallow system without straw retention. The bar with each point indicates the range of the standard error of the mean.

CH_4_ emissions from paddy varied greatly with seasons (Fig. 1). The rate of CH_4_ emissions during the fallow season was very low, ranging from 0.001~0.477 mg m^−2^ h^−1^, with an average rate of 0.057 mg m^−2^ h^−1^ and 0.080 mg m^−2^ h^−1^ for CK-Paddy and S-Paddy, respectively. Seasonal cumulative CH_4_ fluxes were 2.46 and 3.42 kg ha^−1^ for CK-Paddy and S-Paddy, respectively (Table 1). During early rice season, CH_4_ emissions steadily increased after transplantation, reaching peak emissions between the end of May and the beginning of June, with around 13 and 15 mg m^−2^ h^−1^ for CK-Paddy and S-Paddy, respectively (Fig. 1). CH_4_ emissions decreased to about 1 mg m^−2^ h^−1^ at the end of early rice season (Fig. 1). The rate of CH_4_ emissions during early rice season ranged from 0.011~14.962 mg m^−2^ h^−1^, at an average rate of 7.108 mg m^−2^ h^−1^ and 8.824 mg m^−2^ h^−1^ for CK-Paddy and S-Paddy, respectively (Fig. 1). Seasonal cumulative CH_4_ fluxes were 131.55 kg ha^−1^ and 163.07 kg ha^−1^ for CK-Paddy and S-Paddy, respectively (Table 1). During late rice season, CH_4_ emissions increased sharply after transplantation, reaching peak emissions at the end of July, with around 25 and 80 mg m^−2^ h^−1^ for CK-Paddy and S-Paddy, respectively (Fig. 1). Then CH_4_ emissions of S-Paddy decreased greatly to 39 mg m^−2^ h^−1^, whereas CK-Paddy had relatively steady CH_4_ emissions around 20 mg m^−2^ h^−1^, and both decreased to 2~3 mg m^−2^ h^−1^ along with floodwater drainage (Fig. 1). After being re-flooded, CH_4_ emissions gradually increased and reached the next peak emissions which were lower than the previous ones obviously, and CH_4_ emissions gradually decreased to 1~2 mg m^−2^ h^−1^ before harvest (Fig. 1). The rate of CH_4_ emissions during late rice season ranged from 0.110~79.626 mg m^−2^ h^−1^, with an average rate of 10.966 mg m^−2^ h^−1^ and 23.257 mg m^−2^ h^−1^ for CK-Paddy and S-Paddy, respectively. Seasonal cumulative CH_4_ fluxes were 242.13 and 513.50 kg ha^−1^ for CK-Paddy and S-Paddy, respectively (Table 1).

N_2_O emissions from paddy occurred mainly during the fallow season (Fig. 1). N_2_O emissions from paddy varied greatly during the fallow season, ranging from 0~37 μg m^−2^ h^−1^, with an average rate of 4.2 μg m^−2^ h^−1^ and 10.7 μg m^−2^ h^−1^ for CK-Paddy and S-Paddy, respectively (Fig. 1). During the fallow season, straw mulching accelerated N_2_O emissions, and seasonal cumulative N_2_O fluxes were 0.18 kg ha^−1^ and 0.44 kg ha^−1^ for CK-Paddy and S-Paddy, respectively (Table 1). During the rice season, N_2_O emissions were very low, ranging from −3.3~20.2 μg m^−2^ h^−1^, with an average rate of 2.58 μg m^−2^ h^−1^ and 3.25 μg m^−2^ h^−1^ for CK-Paddy and S-Paddy, respectively (Fig. 1). During the rice season, straw retention had negligible effects on N_2_O emissions, and seasonal cumulative N_2_O fluxes were 0.11 kg ha^−1^ and 0.14 kg ha^−1^ for CK-Paddy and S-Paddy, respectively (Table 1).

### CH_4_ and N_2_O emissions with *ex situ* application of rice straw

**Figure 2:**
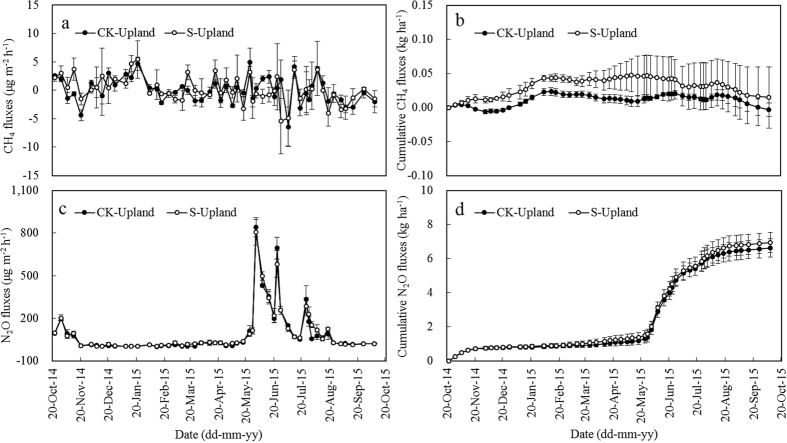
Dynamics of CH_4_ and N_2_O emissions from upland as affected by the *ex situ* application of rice straw. S-Paddy means maize-rape system with straw application at a rate of 5000 kg ha^−1^ season^−1^ and CK-Paddy means maize-rape system without straw application. The bar with each point indicates the range of the standard error of the mean.

CH_4_ emissions from upland were very low throughout the entire year, ranging from −6.4~5.4 μg m^−2^ h^−1^, with an average rate of −0.05 μg m^−2^ h^−1^ and 0.15 μg m^−2^ h^−1^ for CK-Upland and S-Upland, respectively (Fig. 2). Annual cumulative CH_4_ fluxes were −0.005 kg ha^−1^ and 0.013 kg ha^−1^ for CK-Upland and S- Upland, respectively (Table 2).

However, upland soils were important sources of N_2_O. During the oilseed rape season, N_2_O emissions increased to about 200 μg m^−2^ h^−1^ at the first week after base fertilization, then gradually decreased, and kept an average rate of around 10.29 and 15.02 μg m^−2^ h^−1^ for CK-Upland and S-Upland, respectively, during the subsequent over 5 months (from 20-Nov-2014 to 10-May-2015) (Fig. 2). Average rates of N_2_O emissions in the oilseed rape season were 23.77 μg m^−2^ h^−1^ and 27.99 μg m^−2^ h^−1^ for CK-Upland and S-Upland, respectively, and seasonal cumulative N_2_O fluxes were 1.14 kg ha^−1^ and 1.34 kg ha^−1^ for CK-Upland and S-Upland, respectively (Table 2).

During the subsequent maize season, N_2_O emissions increased sharply to more than 800 μg m^−2^ h^−1^ at the first week after base fertilization, and then decreased to about 200 μg m^−2^ h^−1^ during subsequent 25 days (Fig. 2). Similar peak emissions occurred after topdressing at sixth-leaf stage and tenth-leaf stage. Average rates of N_2_O emissions in maize season were 158.33 μg m^−2^ h^−1^ and 162.06 μg m^−2^ h^−1^ for CK-Upland and S-Upland, respectively, and seasonal cumulative N_2_O fluxes were 5.21 kg ha^−1^ and 5.33 kg ha^−1^for CK-Upland and S-Upland, respectively (Table 2). More than 75% of N_2_O fluxes occurred in maize season (Table 2). There were about 50 non-crop days. During the non-crop period, the cumulative N_2_O fluxes were 0.34 kg ha^−1^ and 0.32 kg ha^−1^ for CK-Upland and S-Upland, respectively (Table 2). On the whole, the effects of straw mulching in upland on CH_4_ and N_2_O emissions were insignificant.

### Comparison of GWP between *in situ* application and *ex situ* application of rice straw

The main GHG from paddy was CH_4_ (Table 1). The CH_4_ emission from CK-Paddy plots was equivalent to 9398 kg CO_2_, accounting for 99% of GWP. And the CH_4_ emission from S-Paddy plots was equivalent to 16950 kg CO_2_, accounting for 98.7% of GWP. The CH_4_ emission from upland was approximate to 0, so the main GHG from upland was N_2_O, accounting for almost 100% of GWP (Table 2). The GWP of paddy was far greater than that of upland, with the former being 8.62 times of the latter (Tables 1 and 2). Straw removal from paddy decreased 51.2% of N_2_O emissions and 44.7% of CH_4_ emissions, thus deceasing 44.8% (7693 kg CO_2_-eqv ha^−1^ yr^−1^) of annual GWP (Table 1). From different seasons, there was 1.4% of GWP decrement (107 kg CO_2_-eqv ha^−1^) during fallow season, 10.4% of GWP decrement (797 kg CO_2_-eqv ha^−1^) during early rice season, and 88.2% of GWP decrement (6789 kg CO_2_-eqv ha^−1^) during late rice season (Table 1). Straw application in upland increased GWP by only 91 kg CO_2_-eqv ha^−1^ yr^−1^ (Table 2), which meant that the *ex situ* application of rice straw could decrease GWP by 7602 kg CO_2_-eqv ha^−1^ yr^−1^.

## Discussion

Paddy-upland coexisting systems are widely distributed in hilly areas in subtropical China. In the present study, the *ex situ* straw application of rice straw is different from straw (*in situ*) retention which is one of the principles of conservation agriculture. The *ex situ* application of rice straw can facilitate both nutrition recycling and GHG mitigation. To our best knowledge, few studies report the effects of rice straw *ex situ* application on mitigation of GHG from paddy-upland coexisting system. In this study, we conducted year-round measurements of CH_4_ and N_2_O emissions from a subtropical paddy-upland coexisting system in China, which helped improve our understanding of CH_4_ and N_2_O emissions from subtropical paddy-upland coexisting systems.

Annual cumulative CH_4_ fluxes were 375.9 kg ha^−1^ yr^−1^ and 678.0 kg ha^−1^ yr^−1^ for CK-Paddy and S-Paddy, respectively (Fig. 1), which were within the range of available observations for rice–rice system^7^,^21^,^23^. Consistent with previous studies^7^,^21^,^24^, the rice growing season accounted for the majority of the annual CH_4_ fluxes (>98%, Table 1). Contribution of late rice season (>63%) was more than that of early rice season (<35%), especially when straw was added (Table 1). Moreover, straw retention in fallow and early rice season increased CH_4_ fluxes by 32.7 kg, accounting for 10.7% of annual increment, whereas straw retention in late rice season increased CH_4_ fluxes by 271.4 kg, accounting for 89.3% of annual increment (Table 1). Similarly, Wang *et al*. reported that straw retention in late rice season increased much more CH_4_ emission than in early rice season^25^. Late rice straw was partially oxidized during fallow season before being embedded into soil in early rice season. Tang *et al*. proved that rice straw decomposition in fallow season led to a decrease in CH_4_ emission during the subsequent rice season^19^. In contrast, it was fresh (early rice) straw that was embedded into soil in late rice season. Gaihre *et al*. reported that elevated temperature increased CH_4_ emission, which was further enhanced by the incorporation of rice straw^26^. In the present study, air temperature in late rice season was higher than that in early rice season, and CH_4_ fluxes were positively correlated with air temperature (*r* = 0.731, *p* < 0.01 (CK-Paddy); *r* = 0.641, *p* < 0.01 (S-Paddy)) during rice seasons. As with observations at other study sites^27^,^28^, CH_4_ emissions decreased rapidly during mid-season drainage and rebounded gradually to secondary peak values after being re-flooded in the present study, which was observed in late rice season (Fig. 1). Similar seasonal patterns of CH_4_ emission have been well explained by the fluctuations of soil oxidation-reduction conditions regulated by field water conditions^29^,^30^. Upland system showed slight uptake or release of CH_4_ during total 52 groups of measurements. CK-Upland and S-Upland showed uptake of CH_4_ in 26 groups and 27 groups of measurements, respectively. Although straw mulching slightly increased CH_4_ emissions, which was likely due to high soil moisture under straw mulching, the annual cumulative CH_4_ fluxes were negligible, irrespective of adding straw or not (Fig. 2 and Table 2).

In the present study, annual N_2_O fluxes in paddy system were 0.29 kg and 0.60 kg for CK-Paddy and S-Paddy treatments, respectively, and the fallow season accounted for 62.3% of the annual N_2_O fluxes in the CK-Paddy treatment and 76.9% in the S-Paddy treatment, respectively (Table 1). The drying-rewetting transition along with rainfall in fallow season provided favorable soil conditions for both nitrification and denitrification, which was similar with the conditions of moist intermittent irrigation but without water logging in rice season^31^,^32^. Straw mulching led to favorable soil moisture conditions and supplied abundant organic substrates for denitrification, thus resulting in more N_2_O release^33^. It is well documented that N_2_O emission in rice growing seasons depended greatly on water regime^14^,^31^,^32^,^34^. In current study, fields were continuously flooded in early rice season, and one mid-season drainage was implemented in late rice season. However, N_2_O emissions in rice growing seasons were negligible, irrespective of straw addition (Table 1), much lower than previous estimates^14^,^32^,^34^. In the rice growing seasons, no N_2_O emission peak appeared after nitrogen fertilization, and similar scenario was also found in some reports^7^,^21^,^35^. Strictly anaerobic conditions in flooded paddy is suitable for denitrification, and the major end product of denitrification is N_2_^36^. So to keep fields flooded after the application of nitrogen fertilizers would be helpful for N_2_O mitigation. Also, no N_2_O emission peak appeared during mid-season drainage. Similar scenarios were reported in a few previous studies^31^,^37^. It was reported that huge amount of CH_4_ emission occurred only at soil redox potential lower than approximately −100 mV, while the emission of N_2_O was not significant when soil redox potential was below +200 mV^38^. In the present study, the soil redox potential during mid-season drainage should be within a similar range (eg. −100~+200 mV), which would prevent CH_4_ production and be low enough to encourage N_2_O reduction to N_2_^38^. As opposed to paddy system, upland system was more favorable for N_2_O emissions. Annual cumulative N_2_O fluxes were 6.69 kg ha^−1^ and 6.99 kg ha^−1^ for CK-Upland and S-Upland, respectively (Table 2). Straw mulching tended to increase N_2_O emissions, which was consistent with a conclusion based on 112 scientific assessments^39^. Nitrogen fertilization always led to a N_2_O emission peak (Fig. 2), which is in line with the fact that N fertilization usually induces pulses of N_2_O emission. Cumulative N_2_O fluxes during maize season (5.21~5.33 kg ha^−1^) accounted for 76~78% of annual cumulative N_2_O fluxes (Fig. 2 and Table 2), which was likely due to the favorable conditions of relatively high temperature, high N rate, and high soil moisture^14^,^36^,^40^. In the present study, N_2_O fluxes were positively correlated with air temperature (*r* = 0.375, *p* < 0.01 (CK-Upland); *r* = 0.402, *p* < 0.01 (S-Upland)) through the entire year. The precipitation was 361 mm during the first month of maize season when basal and sixth-leaf stage fertilizer were implemented, which would be beneficial for N_2_O production.

Consistent with previous studies^7^,^21^,^35^,^37^, CH_4_ emission was a major contributor to the total GWP in paddy system, since cumulative N_2_O fluxes were very low (Table 1). On the contrary, N_2_O emission was a major contributor to the total GWP in upland system, since cumulative CH_4_ fluxes were close to 0 in upland system (Table 2), which was in line with most previous studies. In our study, the annual GWP of CH_4_ and N_2_O emissions from S-Paddy was 17178 kg CO_2_-eqv ha^−1^, 0.81 times higher than that from CK-Paddy. The seasonal GWP of CH_4_ and N_2_O emissions from paddy systems in the present study was higher than the mean value of 3757 kg CO_2_-eqv ha^−1^ season^−1^ (75~22237 kg CO_2_-eqv ha^−1^ season^−1^) derived from 116 observations all over the world^41^. The GWP of CH_4_ and N_2_O emissions from S-Upland and CK-Upland were comparable. The GWP of CH_4_ and N_2_O emissions from maize season (taking up 3/4 of annual GWP) was about 1500 kg CO_2_-eqv ha^−1^, which was comparable with the mean value of 1399 kg CO_2_-eqv ha^−1^ season^−1^ (59~5389 kg CO_2_-eqv ha^−1^ season^−1^) derived from 122 observations all over the world^41^.

Measurements of GHG emissions over the entire annual cycle (including crop period and non-crop period) could help determine system-level management effects on GWP. Such investigations, however, are relatively limited^7^,^35^,^40^. To our best knowledge, we presented here for the first time annual GWP of CH_4_ and N_2_O emissions from a paddy–upland coexisting system, based on year-round measurements. The results indicated that the *ex situ* application of rice straw could significantly mitigate the annual CH_4_ and N_2_O emissions on the system-scaled basis. Year-round measurements indicated that removal of early rice straw played an important role in mitigating GWP. Besides, removal of early rice straw is beneficial for land preparation for late rice, resulting in less straw burning. Furthermore, it is dry season when early rice is harvested, and straw mulching can relieve drought and improve soil fertility of rainfed upland^5^,^8^,^16^. Meanwhile, straw nutrition recycling is also important for maintaining paddy soil fertility^5^,^18^,^42^. The recommended straw application is *in situ* application of late rice straw and *ex situ* application of early rice straw with regard to GWP mitigation, nutrient recycling, and labor cost. The measure is of high feasibility when paddy is adjacent to upland.

Whether the recommended strategy is sustainable still needs to be confirmed. Removal of early rice straw will exert an adverse impact on nutrient recycling in the paddy. However, with the increase in straw biomass, land preparation would become increasingly difficult due to straw incorporation. A long-term trial showed that half amount of straw incorporation plus winter green manure reduced chemical fertilizers by about one third^42^. Besides, early rice straw incorporation caused larger amount of CH_4_ emission during late rice season. Climate warming would have potential impact on crop production. Compared with that in the paddy soil, the concentrations of organic carbon and other nutrient elements in the upland soil are much lower. The transfer of straw from paddy to upland may lead to higher nutrient use efficiency according to Liebig’s law of minimum. Previous studies showed that the *ex situ* application of rice straw not only substituted equivalent amounts of chemical fertilizers but also increased soil fertility and crop yield^5^,^8^,^16^.

## Methods

### Field trails

**Figure 3:**
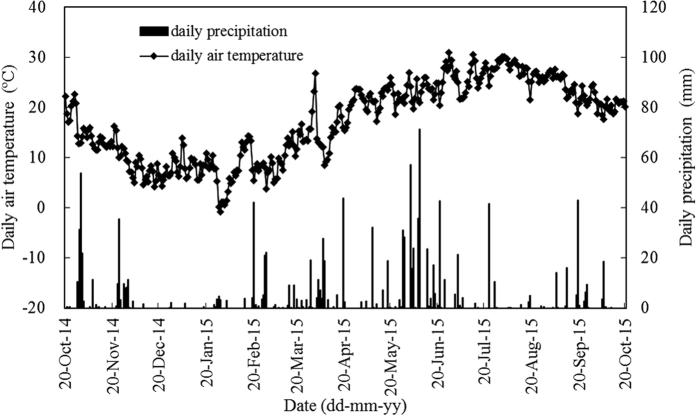
Dynamics of daily precipitation and average air temperature throughout the experimental period.

We chose two adjacent fields, rice-rice-fallow field and maize-rape field, in Taoyuan Station of Agro-ecology Research located in a hilly area with typical paddy-upland coexisting landscapes (28°55′ N, 111°30′ E; altitude: 92.2–125.3 m). The region is characterized by the subtropical humid monsoon climate, with an annual average air temperature of 16.51 °C, precipitation of 1448 mm, sunshine of 1513 h, and frost-free period of 283 days. The soil is developed from Quaternary red clay. The soil properties of the two trials are listed in Table 3. Specific daily precipitation and air temperature during the investigation are shown in Fig. 3.

There were two treatments and three replicates for both rice-rice-fallow system and maize-rape system, respectively. In rice-rice-fallow system, late rice straw was left on soil surface for entire fallow season before being embedded into soil, and early rice straw was embedded into soil immediately (S-Paddy), with the removal of rice straw in both the rice grown seasons as control (CK-Paddy). In maize-rape system, the soil was mulched with rice straw during maize season and rape season (S-Upland), with no straw mulching as control (CK-Upland). Every crop season received the same amount (5000 kg ha^−1^) of rice straw. The fertilizers were applied with urea for N, superphosphate for P and potassium chloride for K. The fertilizing rates were 182 kg N ha^−1^ yr^−1^, 39.3 kg P ha^−1^ yr^−1^ and 198 kg K ha^−1^ yr^−1^ for rice-rice-fallow system, and 341 kg N ha^−1^ yr^−1^, 91.7 kg P ha^−1^ yr^−1^ and 420 kg K ha^−1^ yr^−1^ for maize-rape system. Early rice season received 81 kg N with two splits, 50% as basal fertilizer and 50% as tillering stage fertilizer. Late rice season received 101 kg N with three splits, 50% as basal fertilizer, 33% as tillering stage fertilizer and 17% as panicle fertilizer. Rape season received 101 kg N just as basal fertilizer. Maize season received 240 kg N with three splits, 30% as basal fertilizer, 20% as sixth-leaf stage fertilizer and 50% as tenth-leaf fertilizer. Early rice seedlings were transplanted on April 22, 2015 at a density of 20 cm by 20 cm and harvested on July 8, 2015. Late rice seedlings were transplanted on July 15, 2015 at a density of 20 cm by 25 cm and harvested on October 16, 2015. Rape seedlings were transplanted on October 21, 2014 at a density of 42 cm by 20 cm and harvested on May 10, 2015. Maize seeds were sowed on May 23, 2015 at a density of 56 cm by 36 cm and harvested on September 14, 2015. The early and late rice, maize and oilseed rape used in the experiment were local varieties. The plots of rice-rice-fallow system were flooded during the growing season, except for one drainage during mid-season of late rice. Occasional waterlogging existed due to precipitation during the fallow season. Other aspects of crop cultivation (e.g., sowing and transplanting) and agronomic management (e.g. fertilizing, weed and pest controlling) were performed according to local farming practices.

### Measurements CH_4_ and N_2_O fluxes

The CH_4_ and N_2_O fluxes were measured using a static closed chamber method. Rectangular sampling chamber (60 cm wide × 60 cm long × 100 cm high) was made of sandwich foam plate that could minimize air temperature change inside the chamber when sampling. One 12 volt fan was equipped inside for mixing the gas. In each plot, a chamber-base collar (60 cm wide × 60 cm long) made of polyvinyl chloride plate was permanently fixed into soil at a depth of 15 cm and maintained in place except for tillage. Gas samples were taken from 09:20 am to 10:40 am. Samples of 30 mL gas were taken into pre-evacuated vials with a syringe at 0, 15, 30, 45 and 60 min for each plot to calculate gas change rate. The air temperature inside the chamber was monitored during gas collection. Samples were taken every 6–8 days during crop seasons (except for 3–4 days interval after fertilization), and about every 10 days during fallow season.

The concentrations of CH_4_ and N_2_O were analyzed using a gas chromatograph (Agilent 7890 A, Agilent Technologies, USA) equipped with a flame ionization detector for CH_4_ analyses at 250 °C and using an electron capture detector for N_2_O analyses at 350 °C. CH_4_ and N_2_O fluxes were calculated according to linear change in gas concentration with sampling time, the chamber headspace height, the air pressure and the air temperature within the chambers^21^,^43^. Accumulative CH_4_ and N_2_O emissions were sequentially accumulated from the emissions between every two adjacent intervals of the measurements^31^. Global warming potential (GWP) in a 100-year time horizon was converted into CO_2_ equivalent emissions by multiplying the cumulative emissions of CH_4_ and N_2_O by 25 and 298, respectively^44^.

### Other data measurements

Daily precipitation, air pressure and air temperature were acquired from meteorological station at the research station approximately 200 m from the experimental plots.

### Statistical analyses

All statistical analyses were performed with SPSS 17.0 (SPSS, Inc., USA). The effects of treatments on cumulative CH_4_ and N_2_O fluxes and GWP were assessed using *t* test; *p* < 0.05 was considered statistically significant. The correlation analyses were performed using Pearson correlation analysis.

## Additional Information

**How to cite this article**: Wang, W. *et al*. Mitigating effects of *ex situ* application of rice straw on CH_4_ and N_2_O emissions from paddy-upland coexisting system. *Sci. Rep.* **6**, 37402; doi: 10.1038/srep37402 (2016).

**Publisher’s note:** Springer Nature remains neutral with regard to jurisdictional claims in published maps and institutional affiliations.

## Figures and Tables

**Table 1:** Cumulative CH_4_ and N_2_O fluxes and their estimated global warming potential (GWP) in paddy trial as affected by *in situ* application of rice straw.

Seasons	Treatments	N_2_O kg N_2_O ha^−1^ yr^−1^	CH_4_ kg CH_4_ ha^−1^ yr^−1^	GWP kg CO_2_-eqv ha^−1^ yr^−1^
Fallow season	CK-Paddy	0.18 ± 0.03 b	2.46 ± 0.18 b	116 ± 13 b
	S-Paddy	0.46 ± 0.09 a	3.42 ± 0.19 a	223 ± 23 a
Early rice season	CK-Paddy	0.07 ± 0.01 a	131.4 ± 5.7 b	3304 ± 142 b
	S-Paddy	0.08 ± 0.01 a	163.1 ± 4.2 a	4101 ± 105 a
Late rice season	CK-Paddy	0.04 ± 0.02 a	242.1 ± 9.8 b	6066 ± 240 b
	S-Paddy	0.06 ± 0.02 a	513.5 ± 21.3 a	12855 ± 537 a
Year round	CK-Paddy	0.29 ± 0.05 b	375.9 ± 15.0 b	9486 ± 361 b
	S-Paddy	0.60 ± 0.08 a	678.0 ± 17.3 a	17178 ± 429 a

*Mean ± SE, the letter following each value indicate significance of the difference between values in each column in each period (p < 0.05).

**Table 2:** Cumulative CH_4_ and N_2_O fluxes and their estimated global warming potential (GWP) in upland trial as affected by *ex situ* application of rice straw.

Seasons	Treatments	N_2_O kg N_2_O ha^−1^ yr^−1^	CH_4_ kg CH_4_ ha^−1^ yr^−1^	GWP kg CO_2_-eqv ha^−1^ yr^−1^
Rape season	CK-Upland	1.14 ± 0.20 a	0.01 ± 0.01 a	340 ± 61 a
	S-Upland	1.34 ± 0.26 a	0.05 ± 0.02 a	402 ± 79 a
Maize season	CK-Upland	5.21 ± 0.33 a	0.00 ± 0.01 a	1551 ± 98 a
	S-Upland	5.33 ± 0.45 a	−0.03 ± 0.02 a	1587 ± 134 a
Period without crop	CK-Upland	0.34 ± 0.07 a	−0.01 ± 0.00 a	101 ± 20 a
	S-Upland	0.32 ± 0.05 a	−0.01 ± 0.01 a	96 ± 14 a
Year round	CK-Upland	6.69 ± 0.53 a	0.00 ± 0.01 a	1992 ± 159 a
	S-Upland	6.99 ± 0.60 a	0.01 ± 0.05 a	2083 ± 180 a

*Mean ± SE, the letter following each value indicate significance of the difference between values in each column in each period (p < 0.05).

**Table 3:** Rotation and general soil properties of the paddy and upland trials determined in 2014.

Trials	Rotation	Soil taxonomy	Clay (%)	pH	SOC (g kg^−1^)	N (g kg^−1^)	P (g kg^−1^)	K (g kg^−1^)
Paddy	rice-rice-fallow	Hydragric Anthrosol	32.1	5.6	17.81	1.80	0.586	12.89
Upland	maize-rape	Ultisol	33.5	5.9	10.34	1.24	0.728	13.96
